# The Anti-Virulence Activities of the Antihypertensive Drug Propranolol in Light of Its Anti-Quorum Sensing Effects against *Pseudomonas aeruginosa* and *Serratia marcescens*

**DOI:** 10.3390/biomedicines11123161

**Published:** 2023-11-28

**Authors:** Hadil Faris Alotaibi, Haifa Alotaibi, Khaled M. Darwish, El-Sayed Khafagy, Amr S. Abu Lila, Mohamed A. M. Ali, Wael A. H. Hegazy, Samar Zuhair Alshawwa

**Affiliations:** 1 Department of Pharmaceutical Sciences, College of Pharmacy, Princess Nourah bint Abdulrahman University, Riyadh 11671, Saudi Arabia; 2 Department of Family Medicine, Prince Sultan Military Medical City, Riyadh 12624, Saudi Arabia; 3 Department of Medicinal Chemistry, Faculty of Pharmacy, Suez Canal University, Ismailia 41522, Egypt; 4 Department of Pharmaceutics, College of Pharmacy, Prince Sattam bin Abdulaziz University, Al-kharj 11942, Saudi Arabia; 5 Department of Pharmaceutics and Industrial Pharmacy, Faculty of Pharmacy, Suez Canal University, Ismailia 41522, Egypt; 6 Department of Pharmaceutics, College of Pharmacy, University of Hail, Hail 81442, Saudi Arabia; 7 Molecular Diagnostics and Personalized Therapeutics Unit, University of Hail, Hail 81442, Saudi Arabia; 8 Department of Pharmaceutics and Industrial Pharmacy, Faculty of Pharmacy, Zagazig University, Zagazig 44519, Egypt; 9 Department of Biology, College of Science, Imam Mohammad Ibn Saud Islamic University (IMSIU), Riyadh 11623, Saudi Arabia; 10 Department of Biochemistry, Faculty of Science, Ain Shams University, Abbassia, Cairo 11566, Egypt; 11 Department of Microbiology and Immunology, Faculty of Pharmacy, Zagazig University, Zagazig 44519, Egypt; 12 Pharmacy Program, Department of Pharmaceutical Sciences, Oman College of Health Sciences, Muscat 113, Oman

**Keywords:** propranolol, *Pseudomonas aeruginosa*, *Serratia marcescens*, quorum sensing, bacterial virulence, drug repurposing

## Abstract

The development of bacterial resistance is an increasing global concern that requires discovering new antibacterial agents and strategies. Bacterial quorum sensing (QS) systems play important roles in controlling bacterial virulence, and their targeting could lead to diminishing bacterial pathogenesis. In this context, targeting QS systems without significant influence on bacterial growth is assumed as a promising strategy to overcome resistance development. This study aimed at evaluating the anti-QS and anti-virulence activities of the β-adrenoreceptor antagonist propranolol at sub-minimal inhibitory concentrations (sub-MIC) against two Gram-negative bacterial models *Pseudomonas aeruginosa* and *Serratia marcescens*. The effect of propranolol on the expression of QS-encoding genes was evaluated. Additionally, the affinity of propranolol to QS receptors was virtually attested. The influence of propranolol at sub-MIC on biofilm formation, motility, and production of virulent factors was conducted. The outcomes of the propranolol combination with different antibiotics were assessed. Finally, the in vivo protection assay in mice was performed to assess propranolol’s effect on lessening the bacterial pathogenesis. The current findings emphasized the significant ability of propranolol at sub-MIC to reduce the formation of biofilms, motility, and production of virulence factors. In addition, propranolol at sub-MIC decreased the capacity of tested bacteria to induce pathogenesis in mice. Furthermore, propranolol significantly downregulated the QS-encoding genes and showed significant affinity to QS receptors. Finally, propranolol at sub-MIC synergistically decreased the MICs of different antibiotics against tested bacteria. In conclusion, propranolol might serve as a plausible adjuvant therapy with antibiotics for the treatment of serious bacterial infections after further pharmacological and pharmaceutical studies.

## 1. Introduction

**Figure 1:**
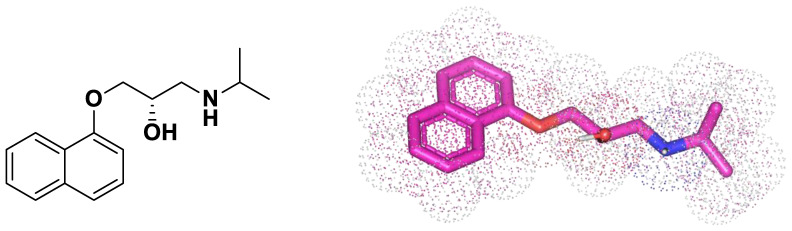
2D- and 3D-structure of the tested *β*-adrenergic receptor blocker, propranolol.

Propranolol is a non-selective β-blocker that is commonly used in the treatment of various cardiovascular conditions (Figure 1). It works by antagonizing epinephrine on the heart and blood vessels thus helping in lowering blood pressure, heart rate, and strain on the heart [1,2]. Additionally, it is also used to manage conditions such as angina, irregular heartbeat, tremors, and to reduce the risk of future heart attacks. Furthermore, propranolol has been prescribed off-label for various conditions, including anxiety, migraine prevention, and essential tremors [3,4]. In our previous leading study, we underscored the applicability of β-adrenoreceptor antagonists, including propranolol, as promising bacterial anti-virulence agents via their possible interfering ability with the bacterial virulence controlling quorum sensing (QS) systems [5]. 

Quorum sensing (QS) systems are the signaling systems that both Gram-positive and -negative bacteria employ to organize their virulence [6,7]. In Gram-negative bacteria, QS systems are working via membranal receptors (QS receptors) that recognize their cognate autoinducers which are produced by synthetases [7,8]. The main autoinducers are acyl-homoserine lactones (AHL) which mainly bind to Lux-type QS receptors in Gram-negative bacteria [7,8,9]. QS systems control the production of diverse virulence factors as well as biofilm formation [9,10,11,12,13]. The crucial role played by QS systems in regulating bacterial virulence renders them valuable targets for mitigating bacterial pathogenesis [9,14]. This approach has been suggested to overcome bacterial resistance development, as mitigating the virulence without affecting the growth could enhance the immunity to remove bacteria without stressing them to develop resistance [15,16,17]. There are several studies that investigate the potential of employing FDA-approved safe drugs as antibacterial agents [18,19,20,21,22,23], natural products [24,25,26,27,28], and several chemical moieties [29,30,31]. Drug repurposing refers to the process of identifying new uses for existing drugs that were originally developed for a different therapeutic indication [32]. Instead of creating a new drug from scratch, researchers explore the potential of existing drugs to treat different diseases or conditions [32,33]. This approach can offer several advantages, including the availability of safety and toxicity data, a known pharmacokinetic profile, and potentially reduced development time and costs compared to developing entirely new drugs [32,33,34]. Of interest, drug repurposing has gained attention as a strategy to find novel antibacterial agents and accelerating their discovery process [33,34,35,36]. Particular attention has been given to the anti-virulence activities of the adrenoreceptor antagonists [5,18,37,38]. 

Bacteria, in particular Gram-negative gut bacteria, can sense a host’s neurotransmitters including adrenergic hormones [39,40,41]. Binding the neurotransmitters to histidine kinase bacterial membranal receptors, the expression of virulence factor encoding genes is enhanced which results in the augmentation of bacterial pathogenesis [41,42,43]. It was suggested that interfering with this bacterial spy on the host cells prevents the exaggeration of bacterial infections [44,45]. Based on the above, propranolol as a β-adrenoreceptor antagonist could confer anti-QS activities and also diminish the adrenergic hormones-induced virulence. This suggests that propranolol’s potential anti-virulence activities can be used in combination with antibiotics [5,20,38]. 

*Pseudomonas aeruginosa* is an aggressive opportunistic Gram-negative rod, and is famed for its ability to induce diverse systematic serious infections [46,47]. Furthermore, *P. aeruginosa* is known by its capability to resist antibiotics and biocides [48]. *Serratia marcescens* is a Gram-negative rod that has gained increasing importance as one of the important nosocomial infectious agents [49,50]. The current study was designed to evaluate the anti-virulence activities of propranolol phenotypically and in vivo against two Gram-negative bacterial models *P. aeruginosa* and *S. marcescens*. Furthermore, the anti-QS activities of propranolol were evaluated virtually and in terms of the expression of QS-encoding genes. 

## 2. Materials and Methods

### 2.1. Minimum Inhibitory Concentrations (MICs) 

The MICs of propranolol were determined against *Pseudomonas aeruginosa* PAO1 and *Serratia marcescens* clinical isolate [51], employing the agar dilution method according to the guidelines of the Clinical Laboratory and Standards Institute Guidelines (CLSI, 2015) [19,52]. To eliminate any potential influence on bacterial growth, bacterial viable counts were performed for overnight cultures of the tested strains at sub-MIC (1/4 MIC) in Luria Bertani Broth (LB) [18,38]. Evaluations of the propranolol anti-virulence activities were conducted at 1/4 MIC.

### 2.2. QS-Encoding Genes Expression

The *P. aeruginosa* RNA extraction was performed from propranolol-treated or untreated cultures at sub-MIC [21]. The used primers to amplify the QS-encoding genes in *P. aeruginosa* have been previously reported [21,51]. The cDNA was synthesized to perform reverse transcription polymerase chain reaction (RT-PCR) quantifying the gene expressions. The relative expressions were calculated using the comparative threshold cycle (2^∆∆Ct^) method in comparison to the *ropD* housekeeping gene [53,54].

### 2.3. Molecular Docking Analysis

Atomic coordinates for the target proteins were downloaded from the RCSB_Protein Data Bank. Using the AutoDocktool package v1.2.0 (Scripps Research Institute, La Jolla, CA, USA), the downloaded protein was structurally prepared through removing the co-crystallized solvent/water/ions, adding the polar hydrogen atoms and partial charges, as well as merging the non-polar hydrogens being missed from the X-ray crystallized PDB file [55]. The prepared target proteins were then converted into PDBQT.file extension to be saved for later use. Synthesized compounds were constructed, converted into 3D-structures, energy minimized at AMBER partial charges/modified forcefield, and then finally converted into PDBQT.files using the OpenBabel tool v.2.3.1 (National Supercomputer Centre, Linköping, Sweden) for subsequent molecular docking protocol [56]. 

Docking workflow was conducted through Lamarckian Genetic Algorithm-driven conformational search under AMBER Forcefield, whereas the Genetic algorithm was adopted for predicting the docked binding modes [55]. Binding sites were defined as endorsing the co-crystallized ligands and/or highlighted using the CASTp 3.0 server as previously mentioned by our research group [37,57]. Docking parameters were set at binding poses of 20, global search exhaustiveness being defined at 100, and maximum energy differences between poses of 4 Kcal/mol [58]. The software provided docked binding scores as free-binding energies (Δ*G*; Kcal/mol). Selecting the best ligand’s docking pose was a combination of considering the higher docking scores, RMSD below 2.0 Å cut-off in relation to the co-crystallized ligand, and/or significant interactions with reported key pocket-binding residues. PyMol v2.0.6 (Schrödinger, New York, NY, USA) was used for visualizing the docking findings and analyzing the ligand-target binding interactions.

### 2.4. Biofilm Inhibition Assay

*P. aeruginosa* PAO1 and *S. marcescens* isolates were reported as strong biofilm-forming strains [18,21,57]. The crystal violet method was used to quantify the biofilm formation in the presence of propranolol at sub-MIC [59,60]. Briefly, 10 μL of tested bacterial suspensions were optically adjusted to OD600 of 0.4 and added to 1 mL of fresh tryptic soy broth (TSB) provided with propranolol at 1/4 MIC. Aliquots of 200 μL with or without propranolol at sub-MIC were transferred to microtiter plates and incubated at 37 °C overnight. The planktonic unadhered bacterial cells were washed out and the adhered cells were fixed with methanol and stained with crystal violet. After washing the excess crystal violet, glacial acetic acid was used to extract the crystal violet which stained the adhered biofilm forming bacterial cells. Finally, the absorbance of the extracted crystal violet was measured.

### 2.5. Assay of Virulence Factors

#### 2.5.1. Bacterial Motility

The swarming bacterial motility was measured on the Müller-Hinton (MH) agar plates supplemented with or without propranolol at sub-MIC [18,37]. Briefly, 5 µL of fresh bacterial cultures were centrally stabbed on the Mueller–Hinton plates and supplemented with or without propranolol at sub-MIC. After overnight incubation at 37 °C, the swarming zones were measured in mm. 

#### 2.5.2. Protease Assay

The extracellular produced proteases were assessed in the supernatants of the overnight cultures using the skim milk agar method [19,57]. The tested bacteria were overnight cultured at 37 °C in the presence or absence of propranolol at sub-MIC, and the supernatants containing the produced extracellular enzymes were collected by centrifugation. Fifty µL of the collected supernatants were transferred to pre-formed wells in 5% skim milk LB agar. After overnight incubation, the diameter of formed clear zones were measured in mm. 

#### 2.5.3. Hemolysins Assay

The hemolysins in the supernatants of fresh overnight bacterial cultures were assayed using fresh 2% erythrocyte suspensions in comparison to completely hemolyzed blood by sodium dodecyl sulfate (positive control) and negative control of non-hemolyzed blood [18]. The collected supernatants (500 µL) in the previous assay were mixed to 1 mL fresh erythrocytes suspension, incubated at 37 °C for 2 h, and then centrifuged. The absorbance of the hemolyzed erythrocytes was measured at 540 nm and compared to positive and negative controls. 

#### 2.5.4. *P. aeruginosa* Pyocyanin Assay

The absorbances of the produced pyocyanin in the *P. aeruginosa* cultures supplied or not with propranolol at sub-MIC were assayed calorimetrically [25]. After incubation at 37 °C for 48 h, LB broth *P. aeruginosa* cultures treated or not with propranolol at sub-MIC were centrifuged and the production of pyocyanin was quantified spectrophotometrically at 691 nm in the supernatants. 

#### 2.5.5. *S. marcescens* Prodigiosin Assay

The produced prodigiosin in the cultures of treated or untreated *S. marcescens* with propranolol at sub-MIC was extracted with acidified methanol and assayed calorimetrically [24]. *S. marcescens* cultures were grown overnight in the presence or absence of propranolol at sub-MIC. The bacterial cells were collected by centrifugation, and acidified ethanol (4% 1M HCl in ethanol) was used to extract prodigiosin. The absorbance of extracted dye was measured at 534 nm.

### 2.6. Determination of the Impact on Potency of the Combination with Antibiotics

The broth dilution method was employed to determine the MICs of antibiotics against *P. aeruginosa* or *S. marcescens* according to CLSI, 2015 [57]. The modified checkerboard method was used to evaluate the effect of propranolol at 1/4 MIC when combined with the tested antibiotics [61,62]. The fractional inhibitory concentration (FIC) (MIC antibiotic in combination/MIC antibiotic alone) was used to assess the output of the combination. The optical densities (OD600) were measured after incubation for 24 hrs. The antagonism was considered when FIC > 4, the synergism was considered when FIC ≤ 0.5, and the indifferent effect was considered when FIC = 0.5 to 4 [57]. 

### 2.7. In Vivo Mice Protection

The propranolol’s mice protection assay against *P. aeruginosa* or *S. marcescens* was conducted [21,25]. Six mice groups were recruited wherein each comprised five *Mus musculus* albino mice. Two groups were intraperitoneally injected with untreated *P. aeruginosa* or *S. marcescens*, as positive groups. The other two groups were not injected or injected with sterile PBS. The last two groups were injected with *P. aeruginosa* treated with propranolol at sub-MIC or *S. marcescens* treated with propranolol at sub-MIC, as test groups. The mice deaths were recorded over five successive days, and the results were plotted using the Kaplan–Meier method. 

### 2.8. Statistical Study

The statistical significance was assured by Student’s *t*-test. All experiments were conducted in triplicate, and the means ± SEM were calculated. The significance was assumed when *p* value < 0.05.

## 3. Results and Discussion 

### 3.1. The MICs of Propranolol and Influence on Bacterial Growth

**Figure 2:**
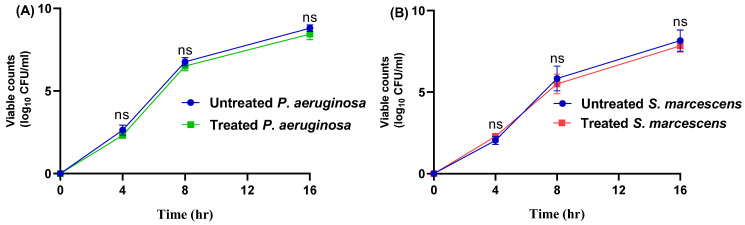
Propranolol at sub-MIC has no significant effect on the growth of (**A**) *P. aeruginosa* or (**B**) *S. marcescens*. There were no significant differences between the viable counts in the presence or absence of propranolol, non-significant (ns): *p* > 0.05.

The MICs of propranolol against *P. aeruginosa* or *S. marcescens* were 2 and 1 mg/mL, respectively. The main principle of bacterial virulence targeting is to alleviate the bacterial virulence to facilitate their eradication by immunity without stressing the bacteria to develop resistance [17,63,64]. This can be achieved by employing the sub-MICs to mitigate the virulence [5,37]. The viability of the growth of the tested strains was evaluated in the presence or absence of propranolol at sub-MICs (Figure 2). There were no significant differences between the viable counts of the tested strains in the presence or absence of propranolol at sub-MICs. 

### 3.2. Propranolol Downregulates the Expression of QS-Controlling Genes

**Figure 3:**
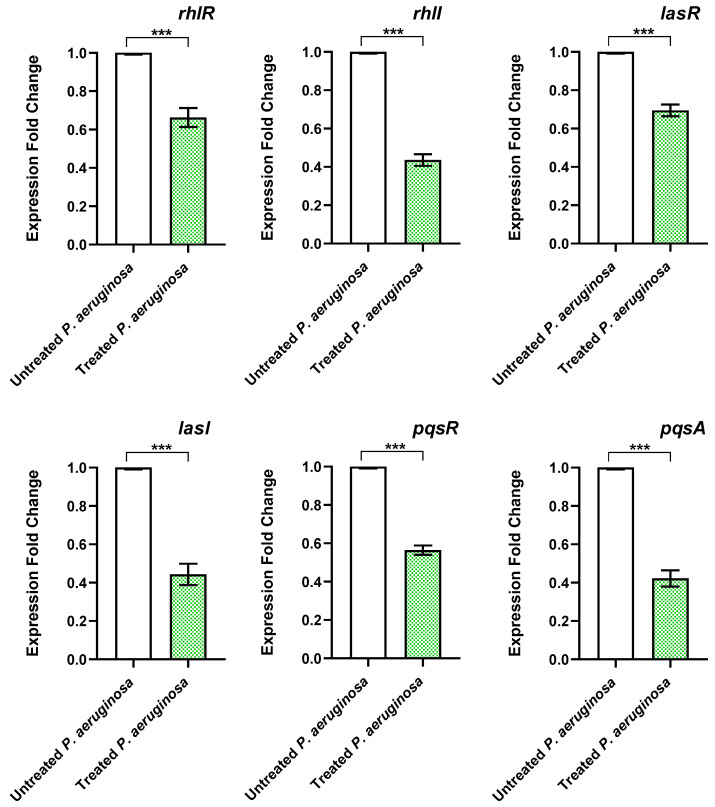
Sub-MIC of propranolol downregulates the expression of QS genes. RT-qPCR revealed the significant downregulation of the QS-encoding genes in *P. aeruginosa*. The expressions of the genes were normalized to the housekeeping gene *ropD.* ***: *p* value < 0.001.

Regulating bacterial virulence is a complex multifaceted process, as the production of virulent factors can vary depending on the stage of infection [64,65,66]. This necessitates diverse sensible systems to orchestrate the production of virulence factors. The QS systems play the key role in controlling the virulence, as the membranal receptors (QS receptors) can sense the produced inducers (autoinducers) in the surroundings [6,9,65,66]. After binding of the inducers and receptors, the formed complex could bind to the upstream sequences of the virulence encoding replicons [5,6]. However, there is quite a difference between the QS systems in Gram-positive and -negative bacteria; the main QS systems in Gram-negative belong to the Lux-type [7,8,67]. For instance, *P. aeruginosa* acquires two Lux-type QS systems, namely the Las and Rhl systems. In addition, QscR is an orphan QS receptor in *P. aeruginosa* that could sense the Las autoinducers. Moreover, *P. aeruginosa* has its own *Pseudomonas* QS (Pqs) system, a non-Lux type, and sense its cognate inducers that are encoded by the *pqsA-D* genes [68]. Similarly, *S. marcescens* employs Sma and Swr QS systems to sense the acyl homoserine lactones autoinducers [50,67]. In the current study, the expression of the genes that encode the main QS receptors in *P. aeruginosa*, *rhlR*, *lasR*, and *pqsR* and their cognate inducers encoding genes *lasI*, *rhlI*, and *pqsA*, respectively, were quantified in the presence or absence of propranolol at sub-MIC. Interestingly, propranolol showed significant reducing effect on the expression of all the QS-encoding genes (Figure 3). These findings could indicate possible anti-QS activity and as a consequence anti-virulence activity of propranolol. 

### 3.3. Propranolol Molecular Aspects towards P. aeruginosa Virulence-Associated Targets 

In order to further explore the anti-virulence activity of propranolol, directed molecular docking investigation was performed against the three main types of *P. aeruginosa* quorum sensing (QS) biotargets. Binding affinity and molecular interactions were investigated for propranolol with the microorganism [i] QS Lux homologs; LasR-type (PDB ID#: 6MVN) [69] and LasI-type (PDB ID#: 1RO5) [70], [ii] non-Lux-type Pqs system; PqsR (MvfR; PDB ID#: 4JVD) [71], and [iii] orphan LuxR homolog; QscR (PDB ID#; 3SZT) [72]. Comparison against reported *P. aeruginosa* QS reference antagonists was performed throughout the presented study for providing a benchmark of biological significance.

#### 3.3.1. Propranolol Binding Affinity towards *P. aeruginosa* QscR

**Figure 4:**
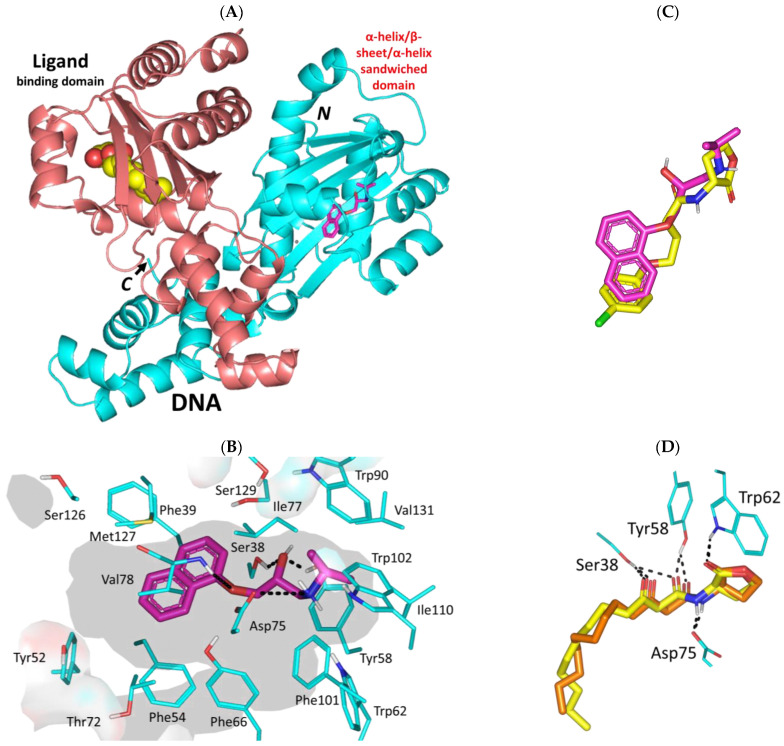
Predicted binding mode of propranolol and docking controls at the *P. aeruginosa* QscR virulence-modulating target. (**A**) 3D cartoon architecture of the dimeric QscR transcription factor (protomers in cyan and orange colors) showing both its DNA binding domain and α-helix/β-sheet/α-helix sandwiched ligand binding domain. Co-crystalline autoinducer, *O*-C12-HSL (yellow spheres), and propranolol (magenta sticks) are shown. Letters *N* and *C* in bold correlate to the amino and carboxy terminals of the protein. (**B**) Zoomed image of propranolol binding pose showing surface representation of the binding site and surrounding residues within 4 Å radius as lines (cyan). Polar interactions, represented as hydrogen bonds, are illustrated as black dashed-lines (**C**) Overlay of both docked propranolol (green) and potent quorum sensing inhibitor chlorolactone (yellow) at the QscR binding site. (**D**) Overlay of co-crystalline *O*-C12-HSL (yellow) and its redocked pose (orange) depicting the same reported orientation/conformation and conserved polar contacts with residues.

Propranolol depicted significant binding affinity at the dimeric QscR’s ligand binding site that comprises the characteristic α-helix/β-sheet/α-helix sandwich shaped domain at the *N*-terminus (Figure 4A). The compound showed deep anchoring at the binding site mediating several hydrophobic interactions with residues lining the almost solvent-inaccessible binding site. Close range hydrophobic interactions with Phe39, Phe54, Ile77, Trp90, Phe101, Trp102, Ile110, Met127, and Val131 have been highlighted via propranolol’s aromatic and aliphatic scaffolds. Notably, sidechains of Phe54 and Val78 served as two arms of a hydrophobic clip providing stability for the in between propranolol’s naphthalene ring via π-π and van der Waal potentials. The ligand’s ionizable amino propranolol arm illustrated a favored direction towards the QscR’s small inner sub-site mediating hydrogen bond interactions with Ser38 (2.4 Å; 133.7°), Tyr58 (2.4 Å; 140.7°) sidechains, Val78 (3.1 Å; 124.5°) mainchain, as well as ionic bond with Asp75 (3.2 Å) (Figure 4B). Interestingly, propranolol showed great superimposition with chlorolactone, a potent synthetic inhibitor of several QS-regulating targets (Figure 4C). Studies in the literature highlight the biological activity of chlorolactone against virulence targets of several microorganisms, including those of *Chromobacterium violaceum*, *P. aeruginosa*, *Agrobacterium tumefaciens*, and *Vibrio fischeri* where this synthetic inhibitor managed to mediate the disassembly of a target’s DNA-binding domains from their respective target genes [16,73,74].

Propranolol was furnished with a higher negative-value docking binding score (−9.92 Kcal/mol) and a wide range of polar interactions as compared to those observed with chlorolactone (−9.27 Kcal/mol). Docking scores were used for predicting *Ki* and ligand efficiency (LE) values being superior for propranolol (*Ki* = 0.06 vs. 0.17 μM; LE = 0.52 vs. 0.46 Kcal/mol), an observation that confers it with a highly preferential competitive activity against the *P. aeruginosa* QscR target. Generally, qualified compounds as leads impose high LE values beyond a threshold cut-off at 0.30 [75,76]. Validation of the docking approach was ensured through redocking the co-crystalline QscR autoinducer, *O*-C12-HSL, depicting great superimposition (aligned RMSD = 1.23 Å), following the same adopted docking parameter and protocols (Figure 4D). Obtaining aligned RMSD values below 2.0 Å typically correlates that both the performed docking algorithm and parameters were efficient for determining the docking pose of potential accuracy [77].

The promising data from the docking studies could indicate the actual propranolol-QscR interactions. Several bisaryl-based molecules with central amide linkers were introduced as anti-Pseudomonal biofilm inhibitors [78,79]. These druggable small molecules showed consistent polar interactions with QscR’s polar residues including Asp75 as well as π-mediated hydrophobic potential forces with Phe54, Tyr66, and/or Trp90. Several antibacterial FDA-approved sulphonamides and their carboxamide-based close analogues depicted favorable polar interactions with QscR’s Tyr58, Asp75, Trp62, and/or Ser129 [80]. Polar interactions with Try58, Asp75, and/or Ser129 were also depicted as important for the QscR-ligand complex stability for a series of triphenyl-based small molecules with antagonistic activity being superior over a QscR’s super-inducer [81]. These aromatic-associated compounds predicted favored face-to-face π-driven interactions with Phe54, Tyr66, Trp63, Trp90, and/or Phe101. Reported in vitro LasR-reporter gene assay findings of these triphenyl-based compounds showed replication of their computational data highlighting their significant antagonistic activity in the presence of a native autoinducer.

#### 3.3.2. Propranolol Binding Affinity towards *P. aeruginosa* LasR

**Figure 5:**
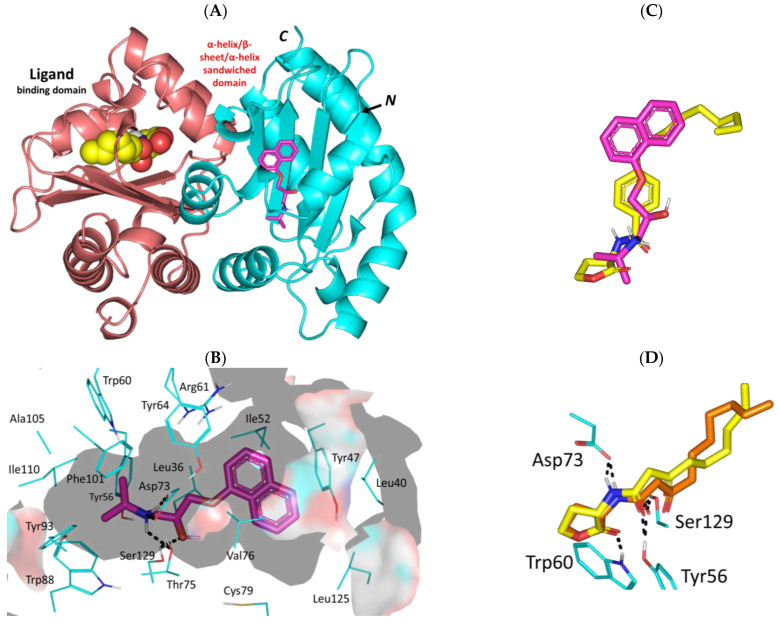
Predicted binding mode of propranolol and docking controls at the *P. aeruginosa* LasR virulence-modulating target. (**A**) 3D cartoon architecture of the dimeric LasR transcription factor (protomers in cyan and orange colors) showing only the α-helix/β-sheet/α-helix sandwiched ligand binding domain. Co-crystalline autoinducer, 3-oxo-C10-HSL (yellow spheres) and propranolol (magenta sticks) are shown. Letters *N* and *C* in bold correlate to the amino and carboxy terminals of the protein. (**B**) Zoomed image of propranolol binding pose showing surface representation of the binding site and surrounding residues within 4 Å radius as lines (cyan). Polar interactions, represented as hydrogen bonds, are illustrated as black dashed lines (**C**) Overlay of both docked propranolol (green) and potent quorum sensing inhibitor, Q9 (yellow), at the LasR binding site. (**D**) Overlay of co-crystalline 3-oxo-C10-HSL (yellow) and its redocked pose (orange) depicting the same reported orientation/conformation and conserved polar contacts with residues.

Moving towards the QS Lux homologs, the investigated β-adrenergic receptor blocker showed preferential anchoring at the LasR’s crystallized ligand binding domain (Figure 5A). The target protein is crystallized in dimeric form of the α-helix/β-sheet/α-helix sandwich-shaped ligand binding domain towards the *N*-terminus. Propranolol managed to achieve deep anchoring at the LasR’s solvent poorly accessible pocket furnishing strong polar interactions with Asp73 (1.8 Å; 147.3°), Thr75 (2.4 Å; 130.0°), and Ser129 (2.4 Å; 129.1°). The ligand’s ionizable amino group served in relevant close-range ionic interaction (2.9 Å) with the Asp73 sidechain. Further ligand stability was translated via both Van der Waal potentials with Leu40, Ile52, Tyr56, Val76, Ala105, Leu110, Leu125, and Phe101, as well as π-driven contacts with Tyr47 and Tyr64. (Figure 5B). Notably, both propranolol and a reported LasR inhibitor, Q9, were depicted at comparable orientation and conformation within the target’s binding site (Figure 5C). We adopted Q9, an aryl-based homoserine lactone derivative, as our positive control ligand since the co-crystallized LasR’s ligand is a cognate autoinducer, 3-oxo-C10-HSL, with relevant gene transcription activation potentiality (EC_50_~0.015 μM). Adopting Q9 was further rationalized since it has been reported with potent antagonism with almost 10-folds superiority over any other LasR inhibitor [82]. Moreover, Q9 failed to possess any atypical partial agonism which is highly characteristic for other LasR inhibitors when applied at higher concentrations [82]. 

Propranolol was furnished with a higher negative-value docking binding score (−10.76 Kcal/mol) and a wide-range of polar interactions as compared to those observed with Q9 (−9.90 Kcal/mol). Docking scores were used for predicting *Ki* and ligand efficiency (LE) values as superior for propranolol (*Ki* = 0.01 vs. 0.06 μM; LE = 0.57 vs. 0.41 Kcal/mol), an observation that confers it with a highly preferential competitive activity against the *P. aeruginosa* LasR target. Validation of the docking approach was ensured through redocking the co-crystalline LasR autoinducer, 3-oxo-C10-HSL, depicting great superimposition (aligned RMSD = 1.06 Å) following the same adopted docking parameter and protocols (Figure 5D). It is worth noting that the depicted propranolol’s target binding pattern was consistent with several LasR inhibitors reported within the current literature. Repurposed FDA-approved drugs as promising *P. aeruginosa* LasR inhibitors depicted consistent polar contacts with Asp73 and Ser129 as well as π-mediated interactions with Tyr64 and Trp88 [83]. Further highlights regarding the significant ligand’s interaction with Asp73 and Ser129 have been reported with the anti-microbial natural flavone, Hispidulin [84]. Combined polar and hydrophobic interaction with LasR pocket lining residues was demonstrated through structure-based virtual screening-coupled molecular dynamic simulations, depicting the significance of polar Tyr56, Asp73, and Ser129 contacts as well as π-π interactions with Tyr47, Tyr64, and Trp88 [85]. These findings highlight the validity of the docking studies to predict the propranolol-LasR binding interactions within the target pocket.

#### 3.3.3. Propranolol Binding Affinity towards *P. aeruginosa* LasI

**Figure 6:**
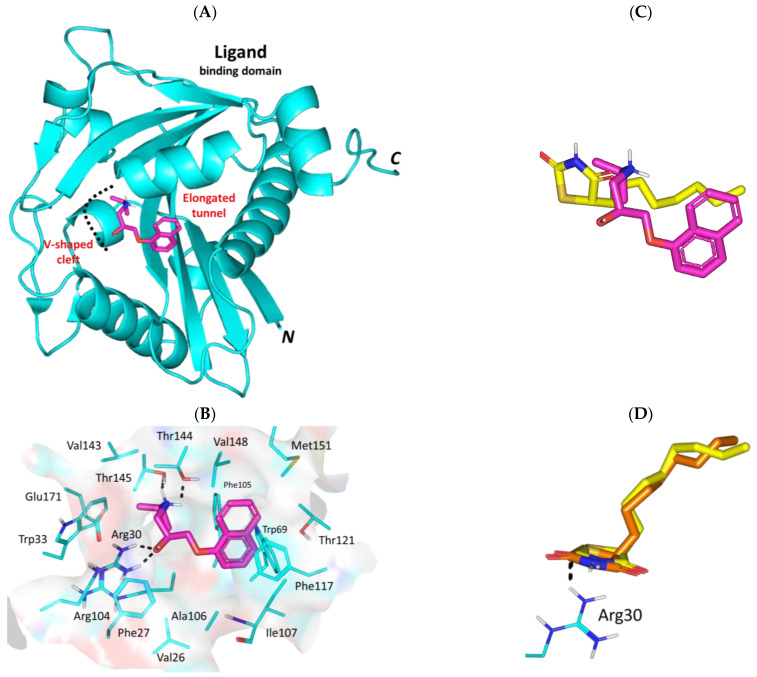
Predicted binding mode of propranolol and docking controls at the *P. aeruginosa* LasI virulence-modulating target. (**A**) 3D cartoon architecture of the monomeric LasI synthase protein (cyan) showing only the ligand binding domain with its V-shaped cleft and elongated tunnel. Docked propranolol (magenta sticks) is shown. Letters *N* and *C* in bold correlate to the amino and carboxy terminals of the protein. (**B**) Zoomed image of propranolol binding pose showing surface representation of the binding site and surrounding residues within 4 Å radius as lines (cyan). Polar interactions, represented as hydrogen bonds, are illustrated as black dashed lines (**C**) Overlay of both docked propranolol (green) and potent LasI inhibitor TZD-C8 (yellow) at the LasI binding site. (**D**) Overlay of docked TZD-C8 (yellow) and its redocked pose (orange) depicting the same reported orientation/conformation and conserved polar contacts with residues.

Molecular docking studies at the other member of the QS Lux homologs revealed favored anchoring of propranolol at the *P. aeruginosa* LasI synthase V-shaped catalytic site. The flexible amino propranolol moiety of propranolol managed to preferentially nest at the pocket’s V-shaped cleft while directing its naphthyl tail towards the pocket’s elongated tunnel (Figure 6A). Stability of propranolol was driven by polar contacts with Arg30 (1.8 Å; 142.6° and 1.9 Å; 139.2°), Thr144 (2.1 Å; 129.8°), and Thr148 (3.2 Å; 124.7°), as well as hydrophobic bonding with Trp33, Ala106, Ile107, Val26, Val143, and Val148. Depicted π-driven interactions with Trp69, Phe105, and Phe117 confer further stabilization for the ligand (Figure 6B). It is worth noting that propranolol oxygen linker functionality was settled at less than 5.0 Å distance from the Ile107 mainchain, a finding that suggests relevant polar interaction through a water bridge. Since the PDB.file of *P. aeruginosa* LasI synthase lacks a co-crystalline native autoinducer, we adopted a reported potent LasI inhibitor, TZD-C8, as our control reference ligand. This thiazolidindione-based positive control was firstly introduced as a synthetic small molecule hampering the LasI-type quorum-sensing signaling synthase of *P. aeruginosa* [86]. Additionally, the same reported study highlighted TZD-C8 as possessing strong inhibitory profiles against biofilm formation and swarming motility, as well as being an efficient inhibitor of the quorum sensing signal production.

To our most delight, both propranolol and TZD-C8 depicted relevant orientations anchoring deep at the V-shaped pocket of LasI (Figure 6C). Despite the more extended and strong interaction patterns depicted with propranolol in relation to TZD-C8, both ligands were assigned with comparable binding scores (−8.62 and −8.59 Kcal/mol, respectively). This could be rationalized to higher flexibility of the TZD-C8’s aliphatic saturated hydrocarbon tail as compared to the flat planner ring within the propranolol structure, a notion that would allow for better orientation and maneuvers for minimal steric energy penalties. Predicted *Ki* and LE values for propranolol and TZD-C8 were also obtained: 0.49 μM, 0.58 Kcal/mol; and 0.52 μM, 0.45 Kcal/mol, respectively. Significance of ligand’s polar interactions with Arg30 and Ile107 have been highlighted in the current literature through site-directed mutagenesis studies where TZD-C8 failed to inhibit LasI signaling following R30D and I107S site mutations [86]. Further validation of the docking protocol was achieved through replicating the TZD-C8 literature which reported interaction patterns and orientations keeping polar interaction with Arg30 (1.8 Å; 123.1°) at the *P. aeruginosa* LasI pocket site [86]. Additionally, the docking protocol was ensured valid for depicting low aligned RMSD (1.09 Å) following a redocking approach for TZD-C8 (Figure 6D). 

#### 3.3.4. Propranolol Binding Affinity towards *P. aeruginosa* PqsR

**Figure 7:**
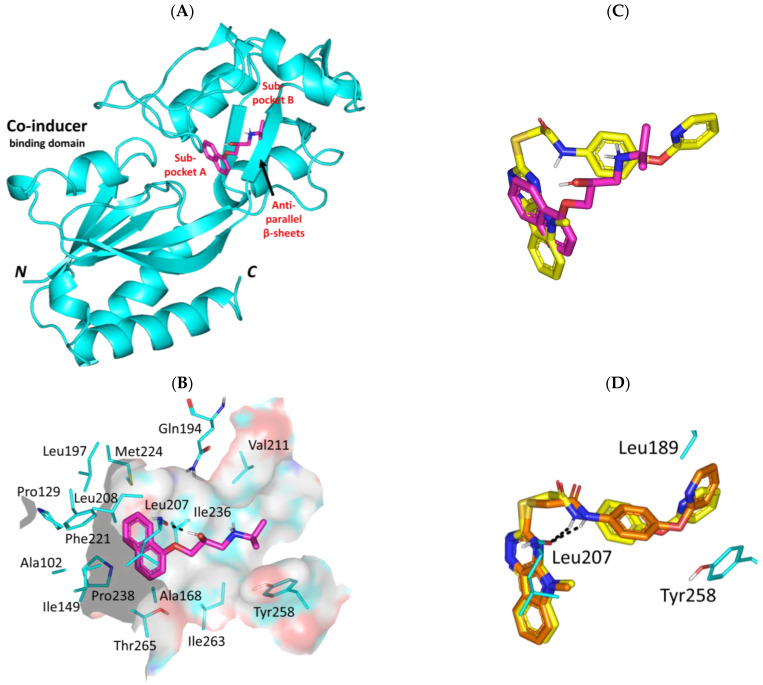
Predicted binding mode of propranolol and docking controls at the *P. aeruginosa* PqsR virulence-modulating target. (**A**) 3D cartoon architecture of the monomeric PqsR synthase protein (cyan) showing only the co-inducer binding domain with its sub-pockets (**A**,**B**) and interconnecting anti-parallel *β*-sheet linker. Docked propranolol (magenta sticks) is shown. Letters *N* and *C* in bold correlate to the amino and carboxy terminals of the protein. (**B**) Zoomed image of propranolol binding pose showing surface representation of the binding site and surrounding residues within 4 Å radius as lines (cyan). Polar interactions, represented as hydrogen bonds, are illustrated as black dashed lines (**C**) Overlay of both docked propranolol (green) and potent PqsR inhibitor NV5 (yellow) at the co-inducer binding site. (**D**) Overlay of co-crystallized NV5 (yellow) and its redocked pose (orange) depicting the same reported orientation/conformation and conserved contacts with residues.

Regarding the non-Lux-type QS, propranolol depicted relevant occupation of the accessible extended hydrophobic pocket of the PqsR co-inducer binding domain. The ligand predicted preferential orientation across both sub-pockets of the binding domain laying its aromatic ring and alkyl amine terminal arm at pockets A and B, respectively. The interconnecting linker of anti-parallel *β*-sheet harbors the hydroxyalkyl scaffold of the docked compound (Figure 7A). Notably, propranolol depicted strong polar contact with Leu207 mainchain (1.9 Å; 165.8°) via its free hydroxyl group granting its stability at the sub-pocket A site. On the other hand, the terminal isopropyl pinned by Val211 and Tyr258 is serving as hydrophobic claws at sub-pocket B with close ranges (4.1 Å and 4.4 Å, respectively) (Figure 7B). Several van der Waals and π-CH hydrophobic contacts were assigned for the ligand’s aromatic ring towards sub-pocket A lining residues: Ile149, Ala168, Leu197, Leu207, Leu208, Phe221, Met224, Ile236, Ala237, and Pro238. 

Overlaying propranolol over the co-crystallized ligand, NV5, showed relevant orientation at the PqsR binding site (Figure 7C). More extended occupation was seen with NV5 for both of the PqsR sub-pockets owing to its larger structure as compared to propranolol. Nevertheless, propranolol managed to be docked at both sub-pockets while maintaining an interaction with Leu207 and Tyr258 being conserved with NV5 and every reported PqsR inhibitor [71,87,88]. Additionally, reported mutagenesis studies, including L207A, L207D, and/or T258A highlighted detrimental activity (~10%) or almost complete activity loss (<2%) for mutant PqsR as compared to the wild-type state [71]. Based on the above depicted docking findings, the differential binding modes have granted propranolol a docking energy, *Ki,* and LE values (−8.71 Kcal/mol; 0.42 μM; 0.46 Kcal/mol) just lower than the co-crystallized ligand (−9.48 Kcal/mol; 0.11 μM; 0.30 Kcal/mol). This is quite promising for propranolol since the reported experimental inhibitory data for NV5 against the Pqs system was at the nanomolar concentrations (IC_50_~0.25 μM) [88]. The adopted docking protocol was ensured valid on depicting low aligned RMSD (1.21 Å) following a redocking approach for the co-crystallized ligand depicting the same reported orientation/conformation and conserved contacts with residues (Figure 7D).

### 3.4. Propranolol Diminishes the Production of Virulence Factors 

QS systems play a pivotal role in the regulation of bacterial virulence, allowing bacteria to assess their population density through the production and detection of signal molecules [89]. Upon reaching a critical threshold, these molecules activate certain genes, triggering coordinated behaviors in the bacterial community [8,9]. These behaviors include the regulation of virulence factors, biofilm formation, and the expression of genes that facilitate survival in host environments [11,50,90]. In order to evaluate the anti-virulence activities and to exclude any effect on bacterial growth, propranolol’s activities were estimated at sub-MIC (1/4 MIC).

#### 3.4.1. Propranolol Diminished Biofilm Formation and Bacterial Motility 

**Figure 8:**
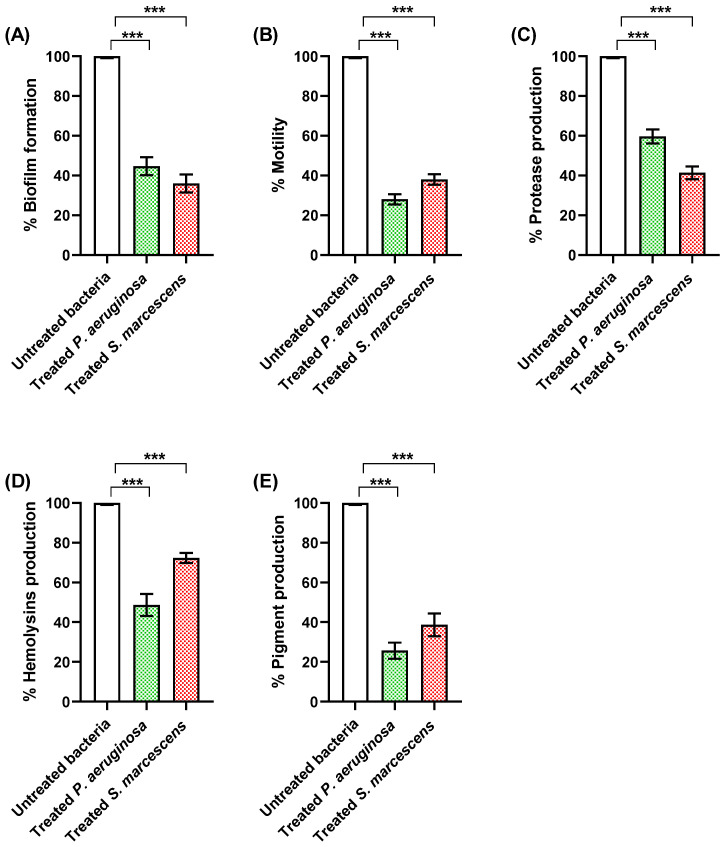
Propranolol at sub-MIC decreased the production of virulence factors in *P. aeruginosa* and *S. marcescens*. Propranolol significantly decreased the (**A**) biofilm formation, (**B**) swarming motility, (**C**) protease production, (**D**) hemolysins production, and (**E**) *P. aeruginosa* pyocyanin pigment and *S. marcescens* prodigiosin pigment. The results are presented as percent change from control untreated bacteria. ***: *p* < 0.001.

The bacterial ability to produce biofilms confers a significant protection against host immunity and enhances resistance to antibiotics [91,92]. The formation of biofilm varies according to the stage of infection that is regulated by the QS systems [11,93]. The formation of biofilms is strongly related to the motility; for instance, the non-motile mutants were unable to form biofilms [59,94]. Inhibition of biofilms is an important target to be achieved by new antimicrobials [6,93]. The current findings clearly inferred the ability of propranolol to diminish biofilm formation (Figure 8A) and interfere with bacterial motility (Figure 8B). 

#### 3.4.2. Propranolol Decreased the Production of Proteases and Hemolysins 

Pathogenic bacteria produce diverse extracellular enzymes that play important roles in the establishment of infections into the host tissues. For instance, proteases and hemolysins enable bacterial spread and overcome the immune system [95,96]. Both *P. aeruginosa* and *S. marcescens* are famed for their ability to produce abundant proteases. Furthermore, *P. aeruginosa* is renowned for its capacity to generate plentiful hemolysins, while *S. marcescens* produces the pore-forming ShlA toxin which causes hemolysis of erythrocytes [97,98]. Propranolol significantly diminished protease production (Figure 8C) and hemolytic activities (Figure 8D) in *P. aeruginosa* and *S. marcescens*. 

#### 3.4.3. Propranolol Decreased the Production of Bacterial Virulent Pigments 

*P. aeruginosa* acquires a huge number of virulence factors and pyocyanin is one of these factors. Pyocyanin is a blue-green, redox-active, and critical component of the pathogenicity of *P. aeruginosa*, contributing to its virulence and pathogenesis [99,100]. Pyocyanin has been associated with various physiological activities, including the generation of reactive oxygen species, interference with host cell signaling, and suppression of the immune response, enabling *P. aeruginosa* to establish and maintain infections in susceptible hosts [99,101,102]. In parallel, prodigiosin is a bright red pigment produced by *S. marcescens*. It is known for its potential biological activities, including antibacterial, antifungal, and immunosuppressive properties [50,103,104]. Prodigiosin has been associated with various pathogenic processes, including the disruption of host cell membranes, induction of apoptosis in eukaryotic cells, and modulation of the host immune response [103,104]. Furthermore, prodigiosin has been implicated in the formation and stabilization of biofilms, which contribute to the persistence of *S. marcescens* in various environments and its resistance to antimicrobial treatments [105,106]. Propranolol significantly decreased the production of *P. aeruginosa* pyocyanin and *S. marcescens* prodigiosin (Figure 8E).

### 3.5. Propranolol Synergistic Effects with Antibiotics

**Figure 9:**
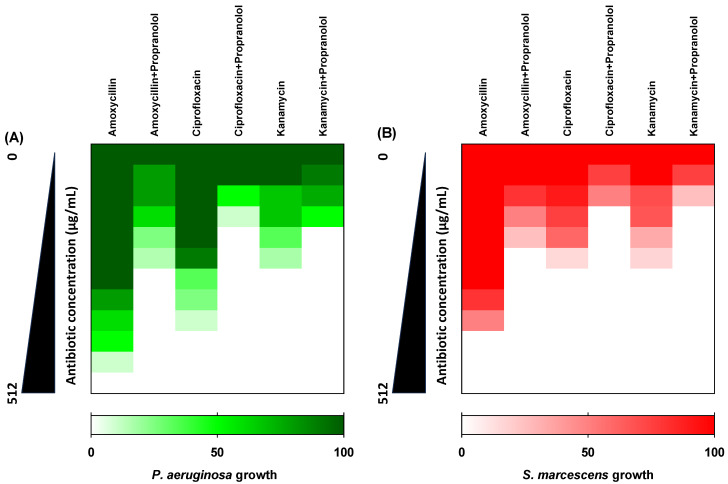
Propranolol at sub-MIC showed synergistic effects when combined with different antibiotics against *P. aeruginosa* or *S. marcescens*.

The checkerboard method was used to evaluate the interactions between three antibiotics that represent three different classes (amoxycillin, ciprofloxacin, and kanamycin) and propranolol at sub-MIC against *P. aeruginosa* and *S. marcescens*. Propranolol effectively synergized the activity of the tested antibiotics showing FIC values ≤ 0.5, indicating significant synergistic activities (Figure 9). 

### 3.6. Propranolol Protected Mice against P. aeruginosa or S. marcescens

**Figure 10:**
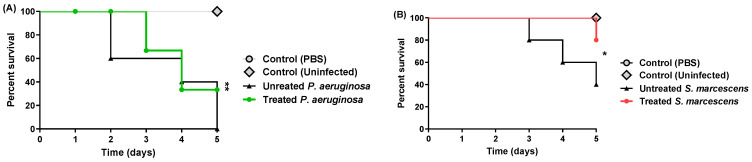
Propranolol at sub-MIC protected mice against *P. aeruginosa* or *S. marcescens*. Propranolol significantly reduced the *P. aeruginosa* or *S. marcescens* capacities to induce pathogenesis (Logrank test for trend *p* = 0.0023 or 0.0407, respectively). *: *p* < 0.05; **: *p* < 0.01.

To conclude the in vivo anti-virulence activity of propranolol against *P. aeruginosa* or *S. marcescens*, the mice protection assay was conducted. Propranolol showed significant protection for mice as it reduced the deaths from five to two in the *P. aeruginosa* treated group (Logrank test for trend *p* = 0.0023) and from three to one in the *S. marcescens* treated group (Logrank test for trend *p* = 0.0407) (Figure 10). These findings indicate the significant ability of propranolol to decrease the capacity of the pathogenic bacteria to induce infections and cause pathogenesis. 

## 4. Conclusions

Propranolol is one of the most frequently used antihypertensive agents and it acquires anti-QS activities. QS systems play the main roles in controlling bacterial virulence and their targeting could assure dramatic decreases in the production of several virulence factors as well as biofilm formation. The anti-virulence activities of propranolol were assessed against two Gram-negative models of *P. aeruginosa* or *S. marcescens*. Propranolol showed significant diminishing activities for all of the tested virulence factors and biofilm formation. Propranolol at sub-MIC diminished the biofilm formation, swarming motility, and production of proteases, hemolysins, and virulent pigments. That could be owed to its anti-QS activities either by binding to the QS receptors or by downregulation of the QS-encoding genes. Furthermore, propranolol significantly lessened the bacterial capacity to induce pathogenesis in mice and showed in vitro synergistic effects when combined with antibiotics. This study suggests the possible employment of propranolol as an adjuvant to antibiotics in treatments of serious infections; however, it requires further pharmaceutical and pharmacological investigations. Moreover, it is encouraging to extend the evaluation of propranolol anti-virulence effects against Gram-positive bacteria and other Gram-negative clinically important pathogens. 

## Data Availability

All the data are published within the manuscript.
